# Acrylamide Content in Breast Milk: The Evaluation of the Impact of Breastfeeding Women’s Diet and the Estimation of the Exposure of Breastfed Infants to Acrylamide in Breast Milk

**DOI:** 10.3390/toxics9110298

**Published:** 2021-11-09

**Authors:** Hanna Mojska, Iwona Gielecińska, Joanna Winiarek, Włodzimierz Sawicki

**Affiliations:** 1 Department of Nutrition and the Nutritive Value of Food, National Institute of Public Health-NIH-National Research Institute, Chocimska 24, 00-791 Warsaw, Poland; 2 Department of Dietetics and Food Studies, Faculty of Science and Technology, Jan Dlugosz University in Czestochowa, Waszyngtona 4/8, 42-200 Częstochowa, Poland; 3 Department of Food Safety, National Institute of Public Health, NIH-National Research Institute, Chocimska 24, 00-791 Warsaw, Poland; igielecinska@pzh.gov.pl; 4 Chair and Department of Obstetrics, Gynecology and Gynecological Oncology of Medical University of Warsaw, Kondratowicza 8, 03-242 Warsaw, Poland; j.winiarek@wum.edu.pl (J.W.); saw55@wp.pl (W.S.)

**Keywords:** breast milk, acrylamide, LC–MS/MS, infants, exposure

## Abstract

Acrylamide in food is formed by the Maillard reaction. Numerous studies have shown that acrylamide is a neurotoxic and carcinogenic compound. The aim of this study was to determine the level of acrylamide in breast milk at different lactation stages and to evaluate the impact of breastfeeding women’s diet on the content of this compound in breast milk. The acrylamide level in breast milk samples was determined by LC–MS/MS. Breastfeeding women’s diet was evaluated based on the 24 h dietary recall. The median acrylamide level in colostrum (n = 47) was significantly (*p* < 0.0005) lower than in the mature milk (n = 26)—0.05 µg/L and 0.14 µg/L, respectively. The estimated breastfeeding women’s acrylamide intake from the hospital diet was significantly (*p* < 0.0001) lower than that from the home diet. We found positive—although modest and borderline significant—correlation between acrylamide intake by breastfeeding women from the hospital diet µg/day) and acrylamide level in the colostrum (µg/L). Acrylamide has been detected in human milk samples, and a positive correlation between dietary acrylamide intake by breastfeeding women and its content in breast milk was observed, which suggests that the concentration can be reduced. Breastfeeding women should avoid foods that may be a source of acrylamide in their diet.

## 1. Introduction

Breast milk is a “golden standard” in infant nutrition. It supplies all the necessary nutrients in amounts covering the demand at a particular stage of the infant’s development. It is also a source of a wide range of immunological components that prevent allergies and protect against infection. The World Health Organization (WHO) recommends exclusive breastfeeding for the first 6 months of the infant’s life, followed by continued breastfeeding as complementary foods are introduced, with the continuation of breastfeeding for 2 years or longer as mutually desired by the mother and infant [1]. The content of individual nutrients and bioactive ingredients is affected by both physiological factors associated with, e.g., the mother’s genotype and health, the duration of the pregnancy, the number of childbirths and lifestyle factors including diet during pregnancy and lactation [2,3]. It needs to be emphasised that chemical contaminants arising from environmental pollution and technological contamination, including so-called processing contaminants, (acrylamide for example), may be relatively easily be transferred to breast milk and pose a risk for the infant’s health.

Acrylamide is classified as a “probable human carcinogen” by the International Agency for Research on Cancer [4], which already concluded in 1994 that despite limited evidence of its carcinogenicity in humans, acrylamide carcinogenicity in animal studies is well documented. It also demonstrates neurotoxic properties and it can be damaging to the central and peripheral nervous system for both experimental animals and people exposed to this compound at work [5,6]. Until the end of the previous century, it was thought that acrylamide did not naturally occur in the environment and was only manufactured as a substrate for the synthesis of polyacrylamides, used, among other things, as industrial and drinking water treatment filter fillers and in the paper, textile and cosmetic industries [4]. Greater interest in acrylamide was sparked in 2002 after it was discovered that it is formed in high-carbohydrate products subject to frying and baking [7,8]. Acrylamide in food occurs mainly as a product of the Maillard reaction between free asparagine and reducing sugars, especially glucose and fructose, under temperatures of more than 120 °C [9,10]. The main source of acrylamide in the human diet are potato products, e.g., crisps and French fries, cereal products, e.g., bread, breakfast cereals, cookies and confectionery, as well as coffee and its substitutes. The estimated mean exposure to acrylamide from food in Europe ranges from 0.4 to 1.4 µg/kg bw/day [11]. Another important source of human exposure to acrylamide is tobacco smoke [11,12,13,14]. In recent years, there has been research into the fate of acrylamide during digestion in the human digestive tract [15] and potential acrylamide formation from other intermediate products of the Maillard reaction, released both during digestion in the digestive tract and coexistent with acrylamide in consumed products—from 5-hydroxymethylfurfural for instance [16,17].

Considering that acrylamide is a genotoxic, carcinogenic and neurotoxic compound, the margin of exposure (MOE) criterium is recommended for its risk assessment [18]. The MOE is the ratio of the Benchmark Dose Lower Limit (BMDL10) to the estimated human intake of the compound. JECFA [19] considered it appropriate to use 0.18 mg/kg bw per day (the lowest value in the range of BMDL10 values) for tumours in the Harderian gland of male mice and 0.31 mg/kg bw per day for mammary tumours in female rats and advises that an MOE of less than 10,000, based on a BMDL10 from animal study, may be of concern for human health. In turn, EFSA [11] calculated a BMDL10 (for a 10% increase in incidence of tumours in a study in mice) of 0.17 mg/kg body weight/day for the carcinogenicity of acrylamide and a BMDL10 of 0.43 mg/kg bw/day for effects on the nervous system in rats. EFSA concluded that possible health risks arising from dietary exposure to acrylamide should be assessed by comparison with the BMDL10 of 0.17 mg/kg bw/day for neoplastic effects and 0.43 mg/kg bw/day for nonneoplastic effects such as neurotoxicity. Acrylamide is rapidly absorbed and owing to its very good solubility in water, rapidly distributed to various tissues. It is metabolised by two main pathways: epoxidation to glycidamide and glutathione conjugation to mercapturic acids. The conversion of acrylamide into glycidamide, its main metabolite, is catalysed by an enzyme of cytochrome P450 (isoenzyme CYP2E1) [20]. Both acrylamide and glycidamide are conjugated to glutathione using S-transferase glutathione enzymes (GST): acrylamide to N-Acetyl-S-(2-carbamoylethyl)-L-cysteine (AAMA) and glycidamide to N-Acetyl-S-(2-carbamoyl-2-hydroxyethyl)-L-cysteine (GAMA) and N-acetyl-S-(3-amino-2-hydroxy-3-oxopropyl)-cysteine. The metabolites of acrylamide and glycidamide in the form of mercapturic acid derivatives are excreted in urine [21]. AAMA and GAMA are considered biomarkers of short-term exposure to acrylamide because it was found that these are already present in urine 2 h after acrylamide intake [22], while their excretion continues for approximately the next three days [23]. On the other hand, adducts to the N-terminal valine of haemoglobin (AA-Hb) are considered biomarkers of long-term exposure [24].

Acrylamide can penetrate the placental barrier [25], thus posing a risk to the growing foetus. In animal studies, the adverse effects of acrylamide consumed with feed during pregnancy on the reproductive performance of animals and the offspring’s developmental parameters [26,27] have been demonstrated. So far, four epidemiological studies confirmed the link between dietary acrylamide intake by pregnant women and small birth weight, the length and head circumference of neonates, or an increased risk of giving birth to an infant who is small for gestational age (SGA) [28,29,30,31]. On the other hand, Nagata et al. [32] did not find inverse association between the dietary acrylamide intake of 204 pregnant women in Japan and the birth size of neonates. At the same time, they found that a higher intake of acrylamide was significantly positively associated with higher levels of umbilical cord oestradiol at delivery [32]. Animal studies [33,34] have shown that giving acrylamide orally to female rats during the lactational period does not induce neurotoxicity or testicular toxicity in the offspring. The authors suggested that this was due to the limited lactational transfer of acrylamide through the blood/milk barrier and the low acrylamide level in milk which is insufficient to initiate toxic changes. Similarly, Pabst et al. [35] tested carry-over acrylamide from cattle feed to bovine milk and demonstrated that acrylamide is quickly degraded and excreted, while its level in raw bovine milk was relatively low.

In studies published to date on the level of acrylamide in human milk, Sorgel et al. [36] evaluated the carry-over of acrylamide in two breastfeeding women who consumed 100 g of potato chips (crisps) with a high content of acrylamide—800 µg/kg and 1000 µg/kg, respectively. The determined acrylamide content in the breast milk samples of the subjects was high and ranged in the first subject from 4.86 µg/L after 4 h to 3.17 µg/L after 8 h from eating potato crisps and, respectively, 10.6 µg/L after 3 h to 18.8 µg/L after 4 h in the other subject. The results indicate that acrylamide is carried over from the mother’s diet to her breast milk and it correlates with the amount of the compound taken in with food. Different results were obtained by Fohgelberg et al. [37] who determined acrylamide in breast milk samples taken from 15 women and in four pooled breast milk samples (10 mothers per pool) in Sweden. They found that in most breast milk samples, the acrylamide content was below the limit of quantification (LOQ = 0.5 μg/kg). Only in one sample, the acrylamide content was above LOQ (0.51 μg/kg). In the author’s opinion, this confirms the fast metabolism of acrylamide in the human body and its limited transfer to breast milk.

The aim of our study was to determine the level of acrylamide in breast milk at two different lactation stages (colostrum and mature milk) and to evaluate the impact of the breastfeeding women’s diet on the acrylamide content in their milk. We also estimated the exposure of infants to acrylamide present in breast milk and the exposure of mothers to the compound present in their diet.

## 2. Materials and Methods

### 2.1. Materials

#### 2.1.1. Study Group

For the women’s recruitment, inclusion and exclusion criteria were essentially the same as described before [38]. In brief, the study group was recruited among healthy women who gave birth in the obstetrics ward of the Chair and Department of Obstetrics, Gynaecology and Gynaecological Oncology at Medical University of Warsaw, during three consecutive months in 2012. The study group inclusion criteria were the mother’s good health, declared exclusive breastfeeding for at least three months postpartum and written consent to participate in the study—including consent to providing a milk sample for testing. The criterion of exclusion were mother’s chronic diseases and child’s genetic defects. From the overall group of 93 women who gave their consent to participation in the study, only 47 women (50.5%) staying in the Obstetrics Ward at 2–3 days postpartum provided a milk (colostrum) sample in an amount sufficient for analysis. During two months of breastfeeding at home, from the initial group of 93 women, a mature milk sample was provided by 26 women (28%). The reasons why the study group became smaller in the 2nd month of breastfeeding include: breastfeeding cessation, inability to contact the subjects (including the address of residence change), and withdrawal from the study without justification. Unfortunately, only 7 women out of the entire group recruited for the study took part at both stages (in hospital and at home).

All participants participated in a sociological interview, which included data such as age, place of residence, education, pregnancy duration. At both stages of the study (in hospital and at home), simple anthropometric measurements of the subjects were performed (height and body weight) and data relating to the infants’ birth weight and length were collected.

All of the procedures performed in the present study were in accordance with the ethical standard from the 1964 Helsinki declaration and its later amendments. Ethical approval was obtained from the Ethics Committee of the National Food and Nutrition Institute in Warsaw, Poland, and the National Science Center (Project No. N N404 067740). Written consent was obtained from all participants.

The characteristics of the breastfeeding mothers and their children are shown in Table 1.

#### 2.1.2. Breast Milk Sample Collection

Colostrum and mature milk samples were taken by the subjects themselves, manually or using a breast pump, and placed in a sterile polypropylene container. Breast milk samples were immediately collected after morning breastfeeding—no less than 15 mL of colostrum and 50 mL of mature milk. If the amount of milk collected at one time was not enough, the milk container was stored in a refrigerator and refilled on the same day after subsequent breastfeeding. The milk samples were delivered under refrigerated conditions to the laboratory as soon as possible, where they were stored at −70 °C until the analysis.

#### 2.1.3. Dietary Intake Assessment

On the same day that a breast milk sample was collected, a face-to-face interview was held regarding food consumption within the past 24 h. The portion size was verified with the use of an “Album of Photographs of Food Products and Dishes” [40].

### 2.2. METHODS

#### 2.2.1. Determining the Acrylamide Level in Breast Milk

##### Chemicals

Deuterium-labelled acrylamide (AAd_3_; 2,3,3-d_3_ acrylamide, 98%) was obtained from Cambridge Isotope Laboratories Inc. (Andover, MA, USA). Acrylamide (AA; 99.5+%) was obtained from Fluka Chemie GmbH (Switzerland). Aluminium potassium sulphate dodacehydrate (KAl(SO_4_)_2_ • 12 H_2_O) for analysis and formic acid (99–100%, GR ACS Reag) LC–MS grade were supplied by Merck KGaA (Darmstadt, Germany). Methanol (HPLC grade, 99.9%) originated from Rathburn Chemicals (Walkerburn, Scotland) and methanol (LC–MS grade, 99.8+) and formic acid (LC–MS grade, 99+%) were from Mallinckrodt Baker BV (Deventer, Holland). ISOLUTE ENV+ (1 g; 6 mL) columns supplied by Biotage AB (Uppsala, Sweden) were used for solid phase extraction (SPE).

Stock reference solutions of acrylamide (0.1 g/L) and deuterium acrylamide (0.1 g/L) were prepared by dissolving 10 mg of each substance in 10 mL of water. Working standards solutions were prepared by diluting the stock solution with water in concentrations of 0.5, 1.0, 2.0, 5.0 and 10 µg/L for AA and 10 µg/L for AAd_3_ (internal standard) with water. All reference solutions were stored at ~4 °C in amber glass vials.

##### Preparation of Breast Milk Samples and LC–MS/MS Analysis

The samples were prepared according to Fohgelberg et al. [37]. In brief, after thawing to room temperature and thorough stirring, 15 mL of milk was collected and 150 μL of the stock reference solution of deuterium acrylamide was added (d_3_-AA; c = 1000 μg/L). The samples were placed in a water bath, then KAl(SO_4_)_2_ • 12 H_2_O was added and the samples were centrifuged and purified on the SPE columns. The supernatant was applied to columns that were previously conditioned with methanol and water. The tested compounds were eluted with 60% methanol solution (LC–MS/MS) and the first 1.7 mL of the eluent was discarded. The collected eluent was concentrated under a nitrogen stream to 1 mL, filtered (0.22 μm PVDF filter) and applied to a chromatographic column.

The acrylamide content in human milk was determined by liquid chromatography coupled with tandem mass spectrometry (LC–MS/MS), as previously described [36]. In brief, chromatographic separation was conducted on a Hypercarb column (150 mm × 2.1 mm, 5 μm, Thermo Scientific, Bellefonte, PA, USA). The analytical conditions were as follows: flow rate—350 μL/min; column temperature—20 °C; injection value—100 μL; mobile phase—mixture of water and LC–MS grade methanol (ratio—9:1, *v*/*v*) with an addition of 0.1% formic acid; runtime—5 min. MS–MS detection was performed in the positive electrospray mode. The multiple degradation patterns *m/z* 72.1 → 55.2 (AA, CE: 14) and *m/z* 75.1 → 58.1 (d_3_-AA, CE: 16) were used for quantification. For verification, the acrylamide degradation pattern *m/z* 72.1 → 44.1 (CE: 20) and *m/z* 75.1 → 47.1 (CE: 14) for d_3_-AA may be used.

The method was validated using the following parameters: selectivity; limit of quantification (LOQ); linearity; working range; within-day precision; between-day precision and recovery.

#### 2.2.2. Estimation of the Exposure of Breastfed Infants to Acrylamide Present in Breast Milk

The exposure to acrylamide in breast milk (µg/kg bw/day) among the group of 73 infants was estimated in the following manner. For 47 neonates (2–3 days postpartum), the results of the acrylamide level determined in the milk (colostrum) of each mother (µg/L), the child’s birth weight (kg) and the infant’s average daily milk consumption appropriate to age (mL) were used to make the estimation. We assumed that a neonate consumes, on average, up to 160 mL of milk per day at 2 days postpartum and 240 mL of milk per day at 3 days postpartum. The average birth weight of the 47 neonates ranged from 2.03 to 4.69 kg (Table 1). For estimating the exposure of infants to acrylamide in breast milk in the 2nd month of life (n = 26), the following data were used: the results of the acrylamide level in the milk of each of the 26 study subjects (µg/L); the body weight of a child in the 2nd month of life at the 50th percentile as read from percentile charts; the infant’s sex considered (Table 1); and an infant’s milk consumption in the 2nd month of life, which, on average, amounts to 720 mL/day [41].

#### 2.2.3. Estimation of the Exposure of Breastfeeding Women to Dietary Acrylamide

The acrylamide intake from hospital and home diets (µg/person/day) by the breastfeeding women and the study subjects’ exposure to acrylamide (µg/kg bw/day) were calculated—taking into account the results of the authors’ own studies on the average acrylamide content in food products in Poland.

The content of acrylamide in each product consumed (in µg/kg) was multiplied by the size of the consumed portion of the product (in kg) calculated based on 24 h recall. For the exposure evaluation, the sum of daily acrylamide intake from all products consumed was divided by the individual body weight (kg) of each study subject. Four of the subjects, at 2nd day postpartum, received only a drip on the day of the test. It included the following infusion liquids: Ringer’s solution, 0.95 NaCl, 5% glucose solution or multi-electrolyte solution, supplied by Fresenius Kabi (Poland). The analytically determined acrylamide content in the above solutions was below the limit of quantification (0.1 μg/L) [38]. For these persons, it was assumed that their acrylamide intake was 0.0 µg/day.

#### 2.2.4. Statistical Evaluation

The description of the physiological and sociodemographic parameters of the study subjects is represented in figures in the form of a median or a percentage of people. The comparison of the above parameters for both study groups was carried out using the χ^2^ test. The acrylamide level in breast milk is expressed in μg/L. The acrylamide intake from diet by the breastfeeding women and the acrylamide intake with breast milk by the infants are expressed in μg/person/day and μg/kg bw/day, respectively. The calculated results were presented as a median and the 95th percentile. To compare the acrylamide intake and exposure at different stages of lactation, the nonparametric Mann–Whitney U test was used. To evaluate the correlation between the acrylamide intake from diet by the breastfeeding women and the level of the compound in their breast milk, Spearman’s correlation coefficient was used. A *p*-value below 0.05 was considered significant for the differences and the dependent correlation. The statistical assessment was carried out using the software Statistica ver. 6.0. (StatSoft Inc., Tulsa, OK, USA).

## 3. Results

### 3.1. The Characteristics of the Breastfeeding Women and Their Children

The characteristics of the participants and their children are shown in Table 1. The majority of the study subjects had university or secondary education and lived in Warsaw. Women from both stages of the study belonged to a similar age group and body weight range. Most of the study subjects had a natural childbirth and for the majority of women, it was their first childbirth. This proportion changed in the home study group where the number of women having their first and the number of women having another baby was similar.

### 3.2. LC–MS/MS Method Validation

The limit of quantification (LOQ) of the method of determining breast milk acrylamide was the concentration of a reference for which the signal-to-noise (S/N) ratio was ≥10. This ratio was determined using the scripts: Savitzky–Goolay Smooth and Signal-to-Noise (Applied Biosystems). The limit of quantification (LOQ) was determined at 0.1 μg/L. For the determination of the acrylamide level in milk, taking into account the expected content of the compound in the samples (evaluated based on the available literature data), an eight-point calibration curve was determined within the range 0.1–15 μg/L, with the constant content of the internal reference of deuterium acrylamide (d_3_-AA) at 10 μg/L. The eight-point calibration curve was linear in the tested range of 0.1–15 μg/L. The curve’s correlation coefficient amounted to 0.9999 (n = 3). In order to verify the stability of the calibration curve, one reference solution with a low and one with a high concentration was added to each analytical batch. The precision of the method was determined by analysing six parallel milk samples. Within the entire curve, the following validation parameters were obtained—precision: RSD < 2.3%; bias ± 1.3%; n = 3; reproducibility: RSD < 5.2%; bias ± 4.4%; n = 6. Relative standard deviation (RSD) amounted to 0.3% (1.93 μg/L). Accuracy of the method, on the other hand, was checked based on the analysis of the recovery of the study compound. The mean recovery amounted to 98.4%.

The results obtained confirmed that the LC–MS/MS method of determining acrylamide in breast milk is specific to the study compound and is characterised by appropriate precision, accuracy and a low limit of quantification.

### 3.3. The Acrylamide Level in Breast Milk

Table 2 shows the acrylamide level in the individual samples of colostrum and mature milk. In nearly 77% of the colostrum samples (36 samples) and more than 42% of the mature milk samples (11 samples), the acrylamide level was below LOQ (0.1 μg/L).

The majority of the remaining breast milk samples did not contain more than 0.5 μg/L of acrylamide. Only in three colostrum samples and one mature milk sample, the analytically determined acrylamide levels were above 0.5 μg/L. The highest acrylamide level in the tested samples of breast milk was 1.0 μg/L. It was found that the median acrylamide level in mature milk was significantly (*p* < 0.05) higher than that for colostrum (0.14 μg/L vs. 0.05 μg/L).

### 3.4. Dietary Acrylamide Intake of Breastfeeding Women

Women staying at the Obstetrics Ward at 2–3 days postpartum had a typical hospital diet, as previously described [38]. The diet mainly included milk and dairy products, boiled and stewed meat and meat products, boiled potatoes and vegetables, rice and cereal products in the form of bread and cereal flakes. Some of the study subjects declared that they additionally ate various kinds of cookies and rusks. The beverages were mainly water, tea, fruit juices and coffee substitutes. The food consumption interview revealed that the study subject’s home diet in the second month of breastfeeding was more diverse compared to the hospital diet. At home, the breastfeeding women, apart from the aforementioned products, ate soups, various kinds of vegetable salads, fried and roasted meat and fish dishes. The home diet also included products and dishes that are a typical source of acrylamide, mainly fried and baked potato products, such as fried potatoes, chips and potato pancakes, as well as flour dishes, such as pancakes. At home, the subjects drank roast and instant coffee. The acrylamide intake from hospital and home diets were calculated, taking into account the results of our own studies on the average acrylamide content in food products in Poland (Table 3).

**Figure 1:**
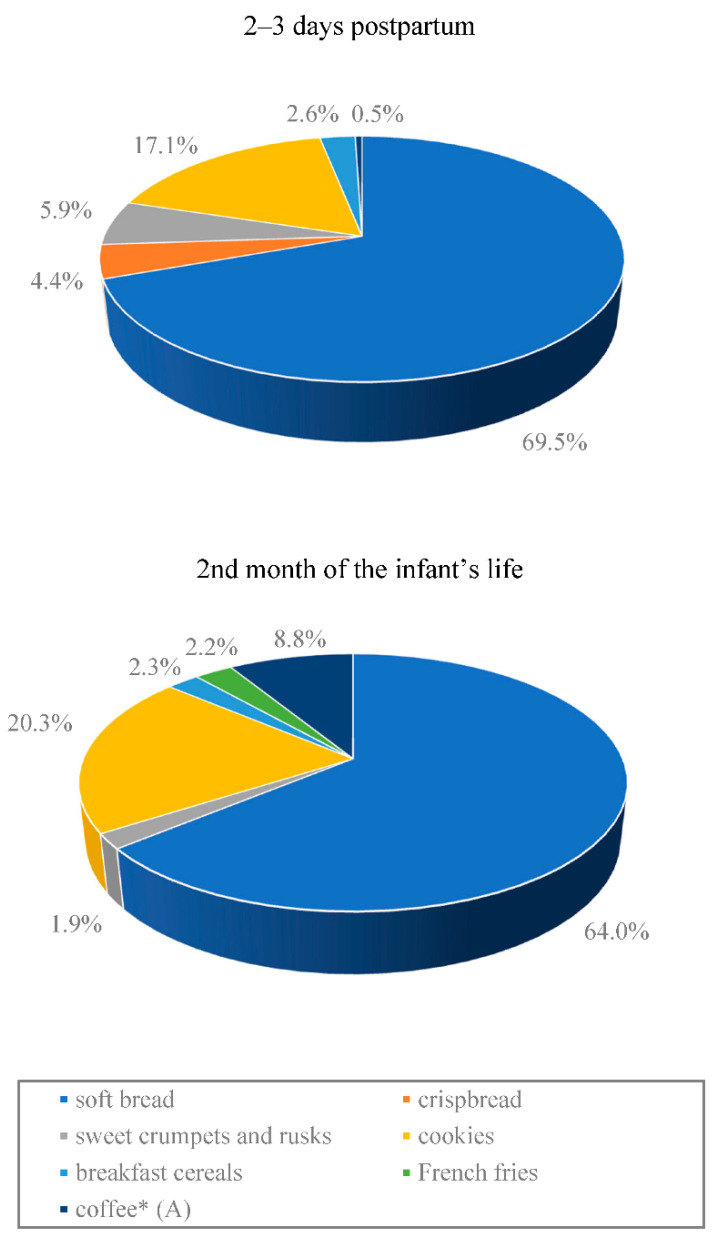
Contribution (%) of the most important food products to dietary exposure to acrylamide in breastfeeding Polish mothers according to the 24 h food consumption data. * Statistically significant difference between the stages of lactation (*p* < 0.05). (A) group ‘coffee’: at 2–3 days postpartum, it was only coffee substitutes, while in the 2nd month of the infant’s life, it was mostly roast and instant coffee.

The average acrylamide intake from the hospital diet, estimated based on 24 h recall, was less than half the intake of the compound from the home diet (7.3 µg/day vs. 16.9 µg/day). The difference was statistically significant (*p* < 0.0001) (Table 4). At both lactation stages, the main source of acrylamide in the study subjects’ diet was bread, followed by cookies (biscuits, rusks, etc.). A significantly (*p* < 0.05) lower intake of acrylamide from coffee only was observed in the group of women staying at the Obstetrics Ward compared to the women on a home diet (Figure 1).

**Figure 2:**
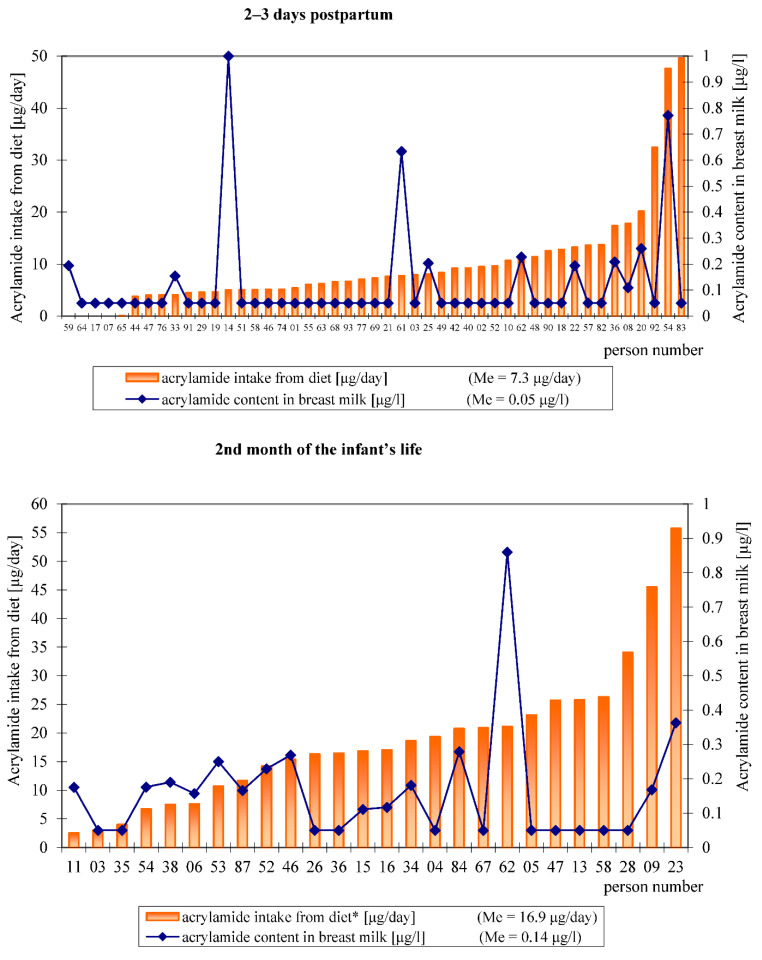
Overview of acrylamide intake by each participant and the acrylamide content in individual breast milk samples.

The distribution of the acrylamide intake from the hospital and home diets by individual study subjects, estimated based on a 24 h recall, relative to the analytically determined acrylamide level in their breast milk is shown in Figure 2.

**Figure 3:**
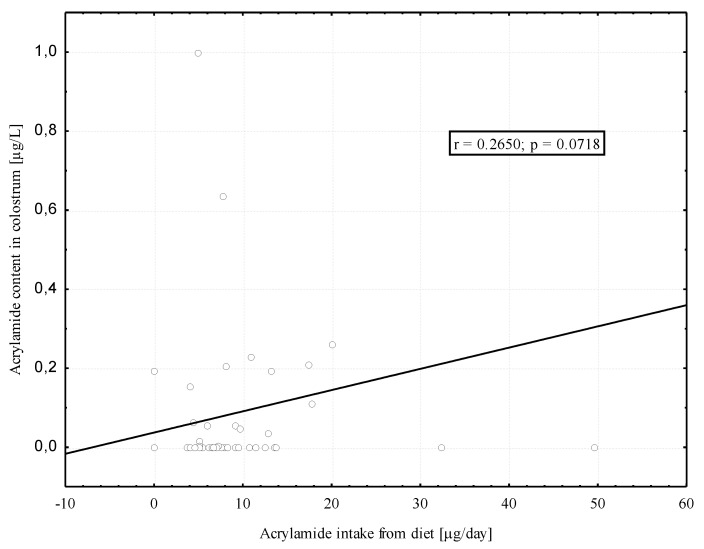
Correlation between the acrylamide intake from diet by the breastfeeding women and the acrylamide in their colostrum.

We found positive, although modest and borderline significant, correlation between acrylamide intake from hospital diet by the breastfeeding women (µg/day) and the acrylamide level in the colostrum (µg/L) (Figure 3). No significant correlation was found between dietary acrylamide intake in the 2nd month of lactation and the acrylamide level in mature milk (r = 0.074; *p* = 0.72).

In the group of seven women who participated at both stages of the study (in the hospital and at home), we did not find a significant correlation between the acrylamide content in colostrum and in the mature milk. There was also no significant association between acrylamide intake from hospital and home diets and between dietary acrylamide intake and its content in breast milk at both stages of lactation.

### 3.5. The Estimation of the Infants’ Exposure to Acrylamide Present in Breast Milk and the Breastfeeding Women’s Exposure to Acrylamide from Their Diet

The estimated exposure to acrylamide from the breastfeeding women’s diet at 2–3 days postpartum was 4.5 times significantly (*p* < 0.0001) lower compared to the group of breastfeeding women in the second month of their child’s life (Table 4). The calculated margins of exposure (MOE) are in most cases below 10,000. The estimated exposure of the newborns to acrylamide in the colostrum was from 4.5 to 6 times significantly (*p* < 0.00001) lower compared to the exposure of the infants breastfed in the second month of life (Table 5). The calculated margins of exposure (MOE) for most infants were higher than 10,000. However, in 5% of infants in the second month of life and 5% of infants on the third day of life, the calculated MOE values were below 10,000.

## 4. Discussion

This was the first study to determine the acrylamide level in breast milk at two different lactation stages: at 2–3 days postpartum (colostrum) and in the second month of breastfeeding (mature milk). The breast milk acrylamide determination results were used to estimate the infants’ exposure to acrylamide present in breast milk and to calculate margins of exposure (MOE) according to JECFA recommendations [19]. We also compared the breastfeeding women’s intake of acrylamide between the hospital diet (2–3 days postpartum) and home diet (second month of breastfeeding) and assessed the impact of the study subjects’ diet on the acrylamide level in their breast milk. Additionally, we estimated the breastfeeding women’s exposure to dietary acrylamide and calculated the margins of exposure (MOE).

Determinations were carried out by liquid chromatography coupled with tandem mass spectrometry (LC–MS/MS). The method validation confirmed that it could be used for determining acrylamide in breast milk as low as 0.1 µg/L. In our study, out of 47 analysed colostrum samples, only in 11 samples (23.4%), the acrylamide level exceeded the limit of quantification (LOQ = 0.1 µg/L). In the case of the mature milk, the acrylamide level exceeding LOQ was found in 15 samples (57.7%). It should be noted that in the majority of the samples, both colostrum (93.6% of the samples) and mature milk (96.1% of the samples), the acrylamide level was not higher than 0.5 µg/L. Only in three samples of colostrum and one sample of mature milk, these values were higher. The highest acrylamide level was observed in one sample of colostrum—it amounted to 1.00 µg/L. The present study found the variability in terms of the acrylamide content, particularly breast milk samples, which was surprising, especially when it comes to colostrum (range from 0.11 to 1.00 µg/L), because the study subjects were on a hospital diet with similar product composition. In addition, four women, on the second day postpartum, received only a drip infusion, where no acrylamide was found [38]. However, in one breast milk sample collected from one of these women, the acrylamide level amounted to 0.20 µg/L, while in the samples of the three remaining women, it was below the LOQ. This could indicate the influence of factors other than diet. It must be noted that Boettcher et al. [22] pointed to the fast acrylamide metabolism, which was evidenced by the occurrence of its metabolites, AAMA and GAMA, in urine already after approximately 2 h from the oral intake of acrylamide. On the other hand, Goempel at al. [23] indicated that acrylamide in the form of AAMA and GAMA was excreted with urine within 3 days from intake, suggesting, at the same time, that endogenous acrylamide formation in the body is possible. Such a possibility was also implied by the study results obtained by Hamzalioglu, A. and V. Gokmen [16,17]. This may explain the variability in colostrum acrylamide level despite having a similar diet (hospital diet). Additionally, individual variations in the rate of acrylamide metabolism cannot be excluded [47]. We also wanted to highlight that that the human milk samples, for ethical reasons, were collected after the baby was fed. Human milk is a water/fat emulsion, but in the final phase of feeding, it contains more fat than in the initial phase, which is dominated by the water fraction [2]. Acrylamide is a very good soluble in water and therefore there may be more of it in the initial phase of breast milk compared to the final phase. It is also worth remembering that babies consume different amounts of milk each time they are fed. This depends on their individual needs. All these factors could influence the variability of the acrylamide content in individual samples.

The acrylamide content in breast milk found in our studies was very similar to the results of Fohgelberg et al. [37], who, studying individual breast milk samples from 15 women and 4 pooled milk samples (milk from 10 women per one sample) in Sweden, observed that the acrylamide level exceeded 0.5 µg/kg in only one milk sample. This value (0.5 µg/kg) constituted a limit of quantification in the cited study. Contrary to our results and those of Fohgelberg et al. [33], Sorgel et al. [36] demonstrated in two breastfeeding women that the breast milk acrylamide level ranged from 3.17 to 18.8 µg/L depending on the person and breast milk sample collection time (3 to 10 h after consumption of acrylamide-containing products). It should be noted that, in our study, and in the Fohgelberg et al. [37] study, participants were on a typical diet. In contrast, in the studies of Sorgel et al. [36], the women purposefully consumed 100 g potato chips (crisps) with a very high acrylamide content of 800 and 1000 µg/kg. The breast milk acrylamide level determined by Sorgel et al. [36] was from ten to several dozen times higher than in our study and the study of Fohgelberg et al. [37]. The results obtained confirm that acrylamide is transferred from the mother’s diet into her milk, indicating at the same time that it is likely to depend on the amount of dietary acrylamide.

In this study, the median acrylamide level in mature milk was significantly (*p* < 0.05) nearly three times as high as the median acrylamide level in colostrum (0.14 µg/L vs. 0.05 µg/L). This seems to confirm, first of all, the impact of diet on the acrylamide level in breast milk. However, further studies are needed to corroborate our finding. A positive correlation, although modest and borderline significant, between the intake of acrylamide from the diet by breastfeeding women and the content in breast milk was only found in relation to colostrum. This is probably due to the small number of women (n = 26) who provided samples of mature milk. Unfortunately, only seven women donated both colostrum and mature milk samples and the results for this group cannot be considered reliable.

Comparing acrylamide intake from hospital and home diets, we found that acrylamide intake from home diet was significantly (*p* < 0.0001) more than twice as high (16.9 µg/day vs. 7.3 µg/day). This is related to the higher consumption of food in general at home compared to the hospital shortly after giving birth, as evidenced by the higher energy value of the home diet (1966 kcal vs. 1395 kcal) and the greater consumption of products that are a source of acrylamide. The obtained results of the median acrylamide intake from diet in the second month of breastfeeding (16.9 µg/day) were slightly lower than our earlier findings [42], where the intake of acrylamide from the diet of adult women in Poland, estimated using a probabilistic approach, averaged 20.4 µg/day. Higher average/median acrylamide intake with the diet in the groups of pregnant women, ranging from 19.6 µg/day in France to 27.1 µg/day in Norway, was presented in the review of Timmermann et al. [48]. This shows that the dietary intake of acrylamide during pregnancy is higher than that during lactation, which was confirmed by our research results and those of Fohgelberg et al. [37]. This seems to indicate a higher exposure to acrylamide in utero than via breastfeeding.

The main source of acrylamide in the diet of breastfeeding women both in the hospital and at home was soft bread, which accounted for approximately 70% of the entire acrylamide intake from diet in terms of the hospital diet and approximately 65% in terms of the home diet. A high daily consumption of various types of bread is specific to the Polish diet. Although it contains low levels of acrylamide, ranging from 13 to 98 µg/kg (except pumpernickel and crispbread), bread invariably is the main source of acrylamide in the Polish diet [38,42]. Significant amounts of acrylamide in the study subjects’ diet were supplied by various types of confectionery products (cookies, biscuits)—17.1% of the entire acrylamide intake from the hospital diet and 20.3% from home diet. Women who breastfed at home introduced coffee into their diets, which accounted for approximately 9% of the entire acrylamide intake from diet. Among all of the products consumed, the only acrylamide intake from coffee was significantly (*p* < 0.05) higher in the group of women breastfeeding in the second month of their child’s life compared to the women at 2–3 days postpartum. It should be noted that the high level of acrylamide in breast milk observed by Sorgel et al. [36], ranging from 3.17 to 18.8 µg/L, depended on the time that elapsed from consumption, the results of the very high acrylamide intake from potato chips (crisps), unseen in our studies. The intake was approximately 5–10 times higher than the average intake estimated in the present study and in the other studies conducted in Europe [37,49]. However, it should be noted that such a high acrylamide content as in potato chips (crisps) described in the study by Sorgel et al. [36] can still occur in various high-carbohydrate products subject to heat processing [11,50]. Therefore, the consumption of significant amounts of products containing acrylamide will result in the transfer of this compound to breast milk and may be associated with adverse effects on the health of breastfed infants. Dietary recommendations for breastfeeding women should include reducing one’s intake of products that can be a source of acrylamide.

The estimated average exposure of the 47 neonates to acrylamide present in breast milk amounted to 0.003 µg/kg bw/day on the second day and 0.004 µg/kg bw/day on the third day of the child’s life. In the second month of breastfeeding, the estimated exposure of the infants (n = 26) to acrylamide in breast milk averaged at 0.018 µg/kg bw/day and was, significantly, (*p* < 0.00001) 4.5–6 times higher compared to that at 3 and 2 days postpartum, respectively. In the present study, a difference was found in the exposure to acrylamide between boys and girls in the second month of life: 0.018 and 0.015 µg/kg bw/day, respectively. However, the difference was not statistically significant. The estimated infants’ exposure to acrylamide present in breast milk was from several dozen to several hundred times lower than in the study by Sörgel et al. [36], in which the authors estimated the breastfed infants’ exposure to be between 0.66 and 3.3 µg/kg bw/day. On the other hand, the results obtained in our study at 2–3 days postpartum (0.03–0.04 µg/kg bw/day) were comparable to the estimated infants’ exposure at 0–6 months of life in Sweden (0.04 µg/kg bw/day) [37]. The infants’ exposure to acrylamide in the second month of life, estimated in our study, was approximately 4.5 times higher than the exposure estimated in the study by Fohgelberg et al. [37].

It should be noted that Fohgelberg et al. [37] did not find significant differences in the acrylamide content between the breast milk and infant formulas on the Swedish market and did not observe a significant difference between the acrylamide intake from breast milk and from infant formulas. In our earlier studies [51], the average acrylamide content in the follow-on formula amounted to 73 µg/kg, and the estimated infant exposure at 6–12 months of life, only with follow-on formula, depending on the month of life and sex, ranged from 0.17 to 1.5 µg/kg bw/day. The breastfed infants’ exposure observed in the present study, depending on the lactation stage, was therefore several to several dozen lower than the exposure from follow-on formulas (0.003–0.018 μg/kg bw/day vs. 0.17–1.5 µg/kg bw/day). It should be noted that, in recently published studies [52], the acrylamide content in products intended for infant nutrition in Turkey was very similar to our findings and averaged at 70 µg/kg, including in infant follow-on formulas, where it averaged 62.5 µg/kg. Additionally, the range of acrylamide content in follow-on formulas was similar. In the study by Basaran and Aydin [52], it ranged from 25.6 to 89.7 µg/kg, while in our own study [51], it ranged from 32 to 78 µg/kg, and in only one sample, reached 312 µg/kg. The results obtained allow us to claim that despite the presence of acrylamide in breast milk, the content of this compound in breast milk is significantly lower than in infant formulas. Additionally, the estimated infant exposure to acrylamide from breast milk is lower than in the case of infant formulas.

According to the JECFA [19], margins of exposure (MOE) of less than 10,000 may be of concern for human health; thus, we calculated the MOE values for neonates, infants and their mothers. The margins of exposure calculated in our study for neonates and infants at the 95th percentile were slightly below 10,000. Therefore, it seems that acrylamide intake from breast milk, as determined in the present study, should not constitute a health risk for most of the infants. However, it needs to be noted that, although the JECFA’s [19] interpretation of calculated MOE values refers to all age groups, it does not specifically refer to neonates and infants. It must be remembered that the neonatal period and infancy differ in terms of the basic vital processes compared to older children and adults. The ability to absorb and assimilate nutrients and to metabolise chemical compounds is different [53]. As research has shown [54], the content of an enzyme of cytochrome P450 (CYP) in the first year of life accounts for approximately 50% of that at the adult level, which could indicate limited CYP activity. It is known that the conversion of acrylamide to glycidamide, its genotoxic metabolite, is catalysed by an enzyme of cytochrome P450 (isoenzyme CYP2E1) [20]. Furthermore, in infancy, the glucuronidation and acetylation of chemical compounds are also limited; however, at the same time, the processes of sulphonation and glutathione-binding are relatively efficient [53]. Although it might seem that these metabolic limitations can have protective effects in infancy when it comes to acrylamide, adverse reactions associated with the still immature metabolic pathways cannot be ruled out.

The present study also estimated the exposure of breastfeeding women to acrylamide present in their diets. An average exposure in the group of women in the second month of breastfeeding amounted to 0.50 µg/kg bw/day and was, significantly (*p* < 0.0001), approximately five times higher compared to the exposure of the women at 2–3 days postpartum (0.11 µg/kg bw/day). The lower exposure in hospital diet arose from the lower consumption of products that can be a source of acrylamide in the diet, but also the overall lower food consumption on the first days after birth. The estimated exposure of women on a home diet to acrylamide was similar to that of the whole Polish population (0.43 µg/kg bw/day), as probabilistically estimated in our previous study [42], and slightly higher than the exposure of adult women in Poland (0.32 µg/kg bw/day) estimated in our study. The average exposure of breastfeeding women on a home diet was also similar to the exposure of pregnant women in Norway—0.44–0.52 µg/kg bw/day [52] and 0.41 µg/kg bw/day [28], respectively, and slightly higher than the exposure of pregnant women in France—0.33 µg/kg bw/day [30]. Despite the relatively small acrylamide intake by the breastfeeding women as compared to that reported by Sorgel et al. [36], both for women on a hospital diet and on a home diet, the calculated average MOE values were considerably below 10,000. The obtained data did not significantly differ from the MOE values calculated for the entire Polish population and particular age groups using a probabilistic approach [42]. The findings of our study show that the exposure to acrylamide in the group of breastfeeding women may be of concern for their health.

## 5. Conclusions

The presence of acrylamide in breast milk at two different lactation stages was confirmed using the LC–MS/MS method. However, in most of the colostrum samples and approximately 50% of the mature milk samples, the level of acrylamide was below the limit of quantification (LOQ = 0.1 μg/L). The positive correlation, although modest and borderline significant, between the acrylamide intake from the diet by breastfeeding women and its content in breast milk (colostrum), indicates that the concentration can be reduced. This requires confirmation in further studies. Despite the presence of acrylamide in breast milk, the content of this compound was considerably lower than in infant nutrition products from various countries, while the exposure arising from the presence of acrylamide in breast milk was also significantly lower than in the case of infants fed with formulas. Apart from the favourable impact of breastfeeding on the infants’ health and development, it is also the best method to protect the young, developing body against processing contaminants in food. However, it should be noted that the calculated margins of exposure for 5% of breastfed newborns and infants indicated that the acrylamide level in breast milk may be of concern for their health. Breastfeeding women should limit the consumption of thermally processed high-carbohydrate products because, if these products are consumed in excessive amounts, the acrylamide present in them may pose a risk for both women and their children.

## Figures and Tables

**Table 1:** Characteristics of breastfeeding women and their children.

Parameters	Median (Range) or Number of People (%)	
**Breastfeeding Women**		
	2–3 days postpartum (n = 47)	2nd month of breastfeeding (n = 26)
Age (years)	31 (21–38)	30 (23–38)
Body weight (kg)	61 (57–96)	60 (50–98)
Education:		
– Primary and vocational	2 (4.3)	–
– Secondary and high school	16 (34.0)	5 (19.2)
– University	29 (61.7)	21 (80.8)
Place of residence:		
– City	43 (91.5)	26 (100)
– Countryside	4 (8.5)	–
Pregnancy duration (weeks):	39 (34–41)	39 (37–41)
Natural birth	27 (57.4)	18 (69.2)
Caesarean section	20 (42.6)	8 (30.8)
Which birth:		
– First	30 (63.8)	12 (46.2)
– Second +	17 (36.2)	14 (53.8)
Energy of all-day diet (kcal/day)	1395 ^a^*^1^ (0 *^2^–2116)	1966 ^b^ (913–2871)
Infants		
	Birth weight (n = 47)	Body weight in the 2nd month of life *^3^ (n = 26)
Body weight (kg):		
– Girls (n = 18/8)	3.13 (2.03–3.76)	5.0
– Boys (n = 29/18)	3.56 (2.10–4.69)	5.6

^a,b^ Median values denoted by different letters statistically and significantly differ. *^1^ Statistically significant difference (*p* < 0.0001). *^2^ For women receiving only the drip infusion on the day of study (n = 4), energy was assumed to be ‘0 kcal/day’ based on the information received from the drip infusion distributor. *^3^ The body weight of children in the 2nd month of life read from percentile charts for the population of children in Warsaw, Poland [39], at the 50th percentile level.

**Table 2:** Acrylamide level in breast milk samples.

Colostrum (2–3 Days Postpartum)		Mature Milk (2nd Month after Birth)	
No. of Participants	Acrylamide Level in Breast Milk (μg/L)	No. of Participant	Acrylamide Level in Breast Milk (μg/L)
01, 02, **03** *^1^, 07, 10, 17, 18, 19, 21, 29, 40, 42, 44, **46**, **47**, 48, 49, 51, **52**, 55, 57, **58**, 63, 64, 65, 68, 69, 74, 76, 77, 82, 83, 90, 91, 92, 93 *^2^	<0.1	**03**, 04, 05, 13, 26, 28, 35, 36, **47**, **58**, 67 *^2^	<0.1
08	0.11	06	0.16
14	1.00	09	0.17
20	0.26	11	0.18
22	0.19	15	0.11
25	0.20	16	0.12
33	0.15	23	0.36
36	0.21	34	0.18
**54**	0.77	38	0.19
59	0.19	**46**	0.27
61	0.63	**52**	0.23
**62**	0.23	53	0.25
		**54**	0.18
		**62**	0.86
		84	0.28
		87	0.17
Me (n = 47)	0.05 *^3^	Me (n = 26)	0.14 *^3^

*^1^ Number of women participating in both studies is marked in bold. *^2^ Limit of quantification LOQ = 0.1 μg/L. For samples whose acrylamide level was < LOQ, half of the LOQ value—0.05 μg/L—was assumed in order to calculate the average value (median, Me). *^3^ Statistically significant difference (*p* < 0.05).

**Table 3:** Acrylamide level in food products consumed by study women *.

Product Group	Acrylamide Level (µg/kg) ^A^	
	Mean	Min ÷ Max
French fries	576	157 ÷ 2175
Wheat–rye bread	31	10 ÷ 99
Rye bread	98	87 ÷ 110
Wholemeal rye bread	43	10 ÷ 108
Graham bread	67	58 ÷ 90
Pumpernickel	190	33 ÷ 430
Wheat rolls	50	23 ÷ 85
Graham rolls	51	27 ÷ 83
Yeast cake and cookies	13	10 ÷ 21
Milk rolls	14	10 ÷ 24
Crispbread	430	65 ÷ 1271
Rusks	20 ^B^	6 ÷ 47 ^B^
Cookies	231	37 ÷ 1178
Crackers	859	566 ÷ 2017
Salty sticks	227	62 ÷ 879
Corn flakes	128	15 ÷ 414
Oat flakes	23	11 ÷ 41
Semolina	1.5 ^B^	–
Crepes	47	43 ÷ 52
Crumpets/pancakes	65	44 ÷ 75
Potato pancakes	1069	650 ÷ 2110
Coffee substitutes (powder)	818	528 ÷ 1145
Roast coffee (powder)	250	61 ÷ 699
Instant coffee (powder)	358	152 ÷ 830

* Data used for estimating the acrylamide intake with diet in breastfeeding women at 2–3 days postpartum and in the 2nd month of lactation; ^A^ source: [42,43,44,45,46]; ^B^ unpublished data.

**Table 4:** Estimated exposure of breastfeeding women to dietary acrylamide and margins of exposure (MOE).

Parameter Tested	2–3 Days Postpartum (n = 47)			2nd Month of Infant’s Life (n = 26)		
	Me	P 95	Min–Max	Me	P 95	Min–Max
Acrylamide intake from diet (μg/person/day)	7.3 ^a^*^1^	32.4	0.00–49.6	16.9 ^b^	45.5	2.5–55.8
Acrylamide exposure (μg/kg bw/day)	0.11 ^a^*^1^	0.41	0.00–0.66	0.50 ^b^	4.84	0.02–5.06
MOE (BMDL_10_ = 0.18 mg/kg bw/day) *^2^	1636	439	273 *^3^	360	37	9000–36
MOE (BMDL_10_ = 0.31 mg/kg bw/day) *^2^	2818	756	470 *^3^	620	64	15,500–61

MOE is the ratio of the Benchmark Dose Lower Limit (BMDL10) to the estimated human intake of the compound [19]. Me—median; P 95—95th percentile. ^a,b^ median values denoted by different letters differ statistically significantly; *^1^ statistically significant difference (*p* < 0.0001); *^2^ source: [19]; *^3^ for max value of acrylamide exposure.

**Table 5:** Estimated exposure of infants to acrylamide in breast milk and margins of exposure (MOE).

Parameter Tested	2–3 Days Postpartum (n = 47)			2nd Month of Infant’s Life (n = 26)		
	Me	P 95	Min–Max	Me	P 95	Min–Max
Acrylamide intake from breast milk *^1^ *^2^ (μg/person/day)	0.008–0.012 ^a^*^3^	0.101–0.152	0.008–0.240	0.099 ^b^	0.261	0.036–0.619
Acrylamide exposure (μg/kg bw/day)	0.003–0.004 ^a^*^3^	0.026–0.040	0.002–0.044	0.018 ^b^	0.047	0.006–0.111
MOE (BMDL_10_ = 0.18 mg/kg bw/day) *^4^	60,000–45,000	6923–4500	90,000–4090	10,000	3830	30,000–16,225
MOE (BMDL_10_ = 0.31 mg/kg bw/day) *^4^	103,333–77,500	11,923–7750	155,000–7045	17,222	6596	51,667–2793

MOE is the ratio of the Benchmark Dose Lower Limit (BMDL10) to the estimated human intake of the compound [19]. Me—median; P 95—95th percentile. *^1^ Acrylamide intake from breast milk was estimated based on individual results of acrylamide content in breast milk; in the case of milk that contained acrylamide at levels below the limit of quantification (0.1 μg/L), half of the limit of quantification, i.e., 0.05 μg/L of milk, was assumed for the purposes of calculations. *^2^ Breast milk consumption: neonates at 2 days postpartum—160 mL/day; at 3 days—240 mL/day; infants in the 2nd month of life—720 mL/day [42]. ^a,b^ Median values denoted by different letters differ statistically significantly. *^3^ Statistically significant difference (*p* < 0.0001). *^4^ Source: [19].

## Data Availability

Not applicable.
